# Zn‐Induced Interactions Between SARS‐CoV‐2 orf7a and BST2/Tetherin

**DOI:** 10.1002/open.202100217

**Published:** 2021-11-17

**Authors:** Maria Petrosino, Francesco Stellato, Roberta Chiaraluce, Valerio Consalvi, Giovanni La Penna, Alessandra Pasquo, Olivier Proux, Giancarlo Rossi, Silvia Morante

**Affiliations:** ^1^ Dipartimento di Scienze Biochimiche “A. Rossi Fanelli” Sapienza Università di Roma Piazzale Aldo Moro 5 00185 Roma Italy; ^2^ Dipartimento di Fisica Università di Roma Tor Vergata and INFN Via della Ricerca Scientifica, 1 00133 Roma Italy; ^3^ INFN - Sezione di Roma Tor Vergata Via della Ricerca Scientifica, 1 00133 Roma Italy; ^4^ CNR - Istituto di chimica dei composti organometallici 50019 – Sesto Fiorentino Italy; ^5^ ENEA CR Frascati Diagnostics and Metrology Laboratory FSN-TECFIS-DIM Via Enrico Fermi, 45 00044 Frascati RM; ^6^ Observatoire des Sciences de l'Univers de Grenoble UAR 832 CNRS Universitè Grenoble Alpes 38041 Grenoble France; ^7^ Centro Fermi – Museo Storico della Fisica e Centro Studi e Ricerche “Enrico Fermi” 00184 Roma Italy

**Keywords:** SARS-CoV-2, orf7a protein, Tetherin/BST2, XANES, Zn speciation

## Abstract

We present in this work a first X‐ray Absorption Spectroscopy study of the interactions of Zn with human BST2/tetherin and SARS‐CoV‐2 orf7a proteins as well as with some of their complexes. The analysis of the XANES region of the measured spectra shows that Zn binds to BST2, as well as to orf7a, thus resulting in the formation of BST2‐orf7a complexes. This structural information confirms the the conjecture, recently put forward by some of the present Authors, according to which the accessory orf7a (and possibly also orf8) viral protein are capable of interfering with the BST2 antiviral activity. Our explanation for this behavior is that, when BST2 gets in contact with Zn bound to the orf7a Cys_15_ ligand, it has the ability of displacing the metal owing to the creation of a new disulfide bridge across the two proteins. The formation of this BST2‐orf7a complex destabilizes BST2 dimerization, thus impairing the antiviral activity of the latter.

## Graphical abstract

**Think zinc**! We conjecture that in the case of SARS‐CoV‐2, the orf7a accessory protein acts as a BST2 antagonist. We provide evidence, based on sequence analysis, Molecular Dynamics simulations as well as XAS experiments, that Zn ions play a key role in the SARS‐CoV‐2 virus strategy to escape the immune response mediated by the BST2 host protein.

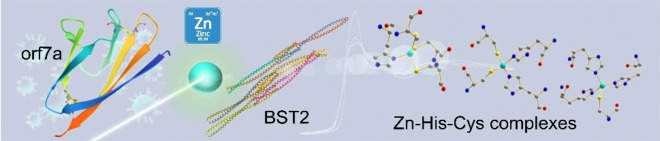

## 1 Introduction

A novel coronavirus of the SARS family, denoted SARS‐CoV‐2, has appeared at the end of 2019[^1^, ^2^, ^3^] and rapidly spread all around the world. A year and a half after its first appearance some 200 million cases of the Covid‐19 syndrome have been reported causing more than 4 million deaths worldwide.^[4]^ Enormous scientific and financial efforts have been invested by researchers, governments, and pharmaceutical companies in the development of safe and effective vaccines. The latter, together with everyday preventive actions, represent at the moment the only way to slow down the pandemic.

National vaccination campaigns for mass immunization have begun, but the aim of finally reaching herd immunity is still very far and may need repeating vaccinations for a number of times. In this situation, it is of the utmost importance to also continue looking for effective antiviral drugs.

One important step in this direction is the identification and characterization of the human proteomes interacting with the SARS‐CoV‐2 proteins. Insert the note „For a review describing the whole set of SARS‐CoV‐2 coded proteins see Ref. [5].” A rather complete and accurate study in this direction has been recently reported^[6]^ and it led to the conclusion that the set of human proteins capable of interacting with the SARS‐CoV‐2 proteins is quite diversified^[7]^ and include a large number of metalloproteins. This situation is shared by other DNA and RNA viruses.^[8]^

It is found that the percentages of Zn, Mg, Fe, Cu, and Mn metalloproteins belonging to the human proteome interacting with the SARS‐CoV‐2 proteins (human SARS‐CoV‐2 metalloproteome) are found to be 12.3 %, 3 %, 1.8 %, 0.3 % and 0.3 %, respectively, with the majority of them (about 17 %) interacting with the accessory protein SARS‐CoV‐2 orf8.^[7]^ As one can see from these numbers, the fraction of Zn‐binding proteins in the human SARS‐CoV‐2 metalloproteome is significantly higher than that of the other metal ions‐binding proteins.

In this context an interesting observation^[9]^ is that several Zn finger motifs, involving cysteine and histidine residues, are present along the SARS‐CoV‐2 accessory orf7a and orf8 proteins.

The orf7a and orf8 proteins (of yet not fully understood function) are coded by the SARS‐CoV‐2 open reading frames ORF7a and ORF8, respectively. The orf7a protein is common to all SARS‐CoV type coronaviruses and is highly conserved,^[9]^ while orf8 is remarkably different from proteins coded by the ORF8 and ORF8b genes of the human SARS‐CoV‐1. The orf7a protein is expressed in the host cell and is likely to be involved in the inhibition of the intracellular (at endoplasmic membrane) process of virion immobilization (tethering), before and after virion vesiculation.^[11]^ On the basis of sequence similarities, it has been argued^[9]^ that also the orf8 protein can be involved in the same process, strengthening the inhibition of the host virion immobilization. The accessory proteins may then have an essential function in the process of immune evasion.^[12]^

The mechanism of immobilization is mainly carried out by proteins of the tetherin family, also known as BST2 or cluster of differentiation 317 (CD317).^[13]^ BST2 is expressed in many cells in the interferon‐dependent antiviral response pathway and is known to be able to block viral replication by trapping enveloped viral progeny on the surface of infected cells, successively leading to virus internalization and degradation.[^14^, ^15^]

**Figure 1:**
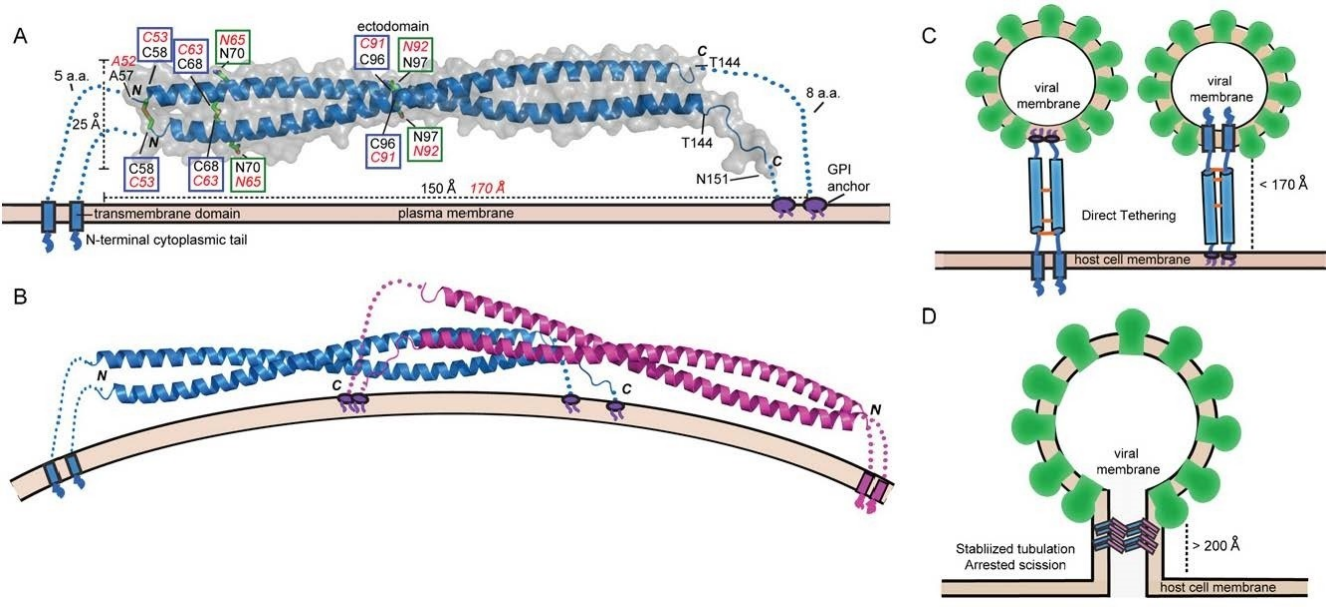
BST2 structure and models of BST2 antiviral action. Panel (A): crystal structure of BST2 ectodomain (code 3NWH[^27^, ^28^] in the RCSB PDB data bank). Panel (B): the dimeric BST2 assembly. Panels (C) and (D): models of BST2‐ mediated inhibition of viral budding. The figure is adapted from Ref. [19].

Three invariant cysteine residues (Cys_53_, Cys_63_, and Cys_91_ in human BST2) stabilize BST2 homodimerization through intermolecular disulfide bonds.^[16]^ At least one cross‐linked cysteine must be present for dimer structural stabilization[^17^, ^18^] (see Figure 1 taken from Ref. [19]). The dimerization process is strongly influenced by divalent cations, with Zn being the most abundant ion, involved in cysteine binding and therefore potentially able to influence disulfide bridge formation.

**Figure 2:**
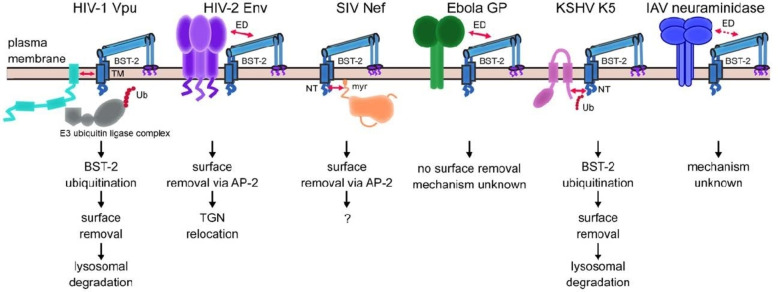
Viral antagonists of BST2 and their mode of action. The cases of HIV‐1^[21,22]^, HIV‐2^[28]^, SIV^[29,30]^, Ebola^[31]^, herpesvirus^[32]^ and IAV^[33]^ are pictorially illustrated. The figure is adapted from Ref. [19].

In Figure 2,^[19]^ the many strategies put in place by different viruses to antagonize the BST2 antiviral activity are pictorially illustrated.

Crystallographic data of the BST2 ectodomain show that the two parallel homodimers are anchored to the host cell membrane.^[20]^ This protein architecture is necessary for antiviral function.[^13^, ^17^, ^18^] Indeed, there is growing experimental evidence that these structural features play a vital role in BST2 antiviral activity. For example, deletion of either one of the membrane anchors makes BST2 ineffective for restricting viral budding.[^18^, ^21^, ^22^, ^23^] On the basis of recent reports[^9^, ^24^, ^25^] it was proposed that cellular Zn control may result in a significant slowing down of viral replication.

An effective way to test these ideas is to study the role of Zn ions in shaping the orf7a and orf8 structure and in the possible formation of BST2/orf7a and BST2/orf8 complexes.

Compared to what happens in the case of other metal ions, where changes in spectral patterns are easily revealed in solution, experimental evidence of Zn binding to proteins is not straightforward, because of the low sensitivity of Zn to changes in the coordination environment when routine spectroscopy (like UV‐Vis) is used. On the other hand, X‐ray Absorption Spectroscopy (XAS) measurements at the Zn K‐edge represents a powerful tool for investigating the near atomic environment of Zn that may allow identifying possible specific Zn‐protein binding sites.

With this in mind, we have performed the first measurements of the entire XAS spectrum of the BST2 protein and the N‐terminal ectodomain of orf7a (PDB 7CI3) at the Zn K‐edge, covering both the X‐ray Absorption Near Edge Structure (XANES) and Extended X‐Ray Absorption Fine Structure (EXAFS) regions, although in the present paper we limit ourselves to comparison, analysis and theoretical calculations of the XANES region of the measured spectra, postponing the study of the EXAFS data to a forthcoming work.^[26]^

Since Cys is a common Zn ligand, we considered especially important to provide experimental evidence for the role played by Cys_15_ of orf7a in the Zn mediated interactions with BST2. For this reason we designed and expressed two orf7a constructs, that we call orf7a_*L*1_ (aa. 16–82) and orf7a_*L2*_ (aa. 15–82), differing for the absence and the presence of the initial Cys_15_. Measurements were performed on the BST2 protein and the two orf7a constructs separately as well as on samples where BST2 and orf7a are simultaneously present.

Of course it would be of the utmost importance to perform the same kind of experiments and analysis by replacing orf7a with orf8. Unfortunately, at the moment we have been unable to produce a sufficiently large amount of the purified orf8 protein, adequate for XAS measurements.

The analysis of the extensive XAS measurements we have performed shows that both BST2 and orf7a are able to bind Zn. We also have indications that the BST2/orf7a complex get formed in the presence of Zn ions, supporting the hypothesis, put forward in Ref. [9], according to which Zn plays a pivotal role in helping to make BST2 available for orf7a binding and inactivation.

These results, besides shedding some light on the selective role of orf7a in the development of the disease could open the way for possible therapeutic strategies against SARS‐CoV‐2 based on the modulation of cellular Zn homeostasis.[^7^, ^9^]

## Experimental Section

Zn is an irreplaceable element in the biochemistry of living cells because of its essential role as a catalytic and structural cofactor.^[34]^ It also seems to have a part in modulating the inhibition of the antiviral BST2 activity by the orf7a and orf8 viral accessory proteins. The recent observation^[9]^ that multiple Zn‐finger domains are present along both the BST2 and the orf7a amino acidic sequence has immediately raised the question of the role of Zn in shaping the structure of these proteins and/or possibly promoting the formation of BST2/orf7a complexes.

As we said, in this context XAS is an ideal tool to study the local Zn environment which is capable of clarifying whether and how Zn can bind to BST2 and/or orf7a.

### Sample Preparation

For the purpose of our XAS experiments we have prepared a number of samples containing either BST2 or the orf7a N‐terminal ectodomain, or the two together in the presence of Zn ions in different solution conditions. For the reasons explained above, we designed and expressed two orf7a constructs, orf7a_*L*1_ (aa. 16–82) and orf7a_*L*2_ (aa. 15–82) that differ for the absence and presence of Cys_15_, respectively. Samples were prepared according to the following protocol.

The pET28a(+) plasmid harbouring the BST2 wild type gene and SARS‐CoV‐2 ORF7a 15–82 or 16–82 genes were synthesized by GenScript^1^. The plasmids were transformed into *E. coli* strain BL21 (DE3) pLySS and expressed overnight at 18 °C after induction with 0.5 mm IPTG (isopropyl‐β‐d‐thiogalactoside, Sigma‐Aldrich, St. Louis, MO) in LB Broth containing kanamycin at a final concentration of 30 μg/mL. Cells were harvested by centrifugation.

Cells derived from BST2 expression were resuspended in a binding buffer (20 mm Tris HCl, pH 8.0, 0.25 m NaCl, 5 mm imidazole and 1 mm Tris (2‐carboxyethyl)phosphine (TCEP)) supplemented with a cocktail of ethylene‐diaminetetraacetic acid (EDTA)‐free protease inhibitors (Sigma‐Aldrich) and lysed by sonication. The lysate was cleared by centrifugation and the soluble fraction was loaded onto a 2 mL prepacked His trap column (GE Healthcare, Chicago, IL) pre‐equilibrated with the binding buffer. The column was washed with the binding buffer to elute weakly bound contaminants and the recombinant protein was eluted by passing over the column binding buffer solutions containing 250 mm imidazole.

Cells derived from SARS‐CoV‐2 ORF7a with 15–82 or 16–82 aa. expressions were resuspended in lysis buffer (50 mm Tris HCl, pH 8.0, 2 mm EDTA, 100 mm NaCl, 1 mm DTT, 0.5 % Triton‐X100) in presence of a cocktail of ethylenediaminetetraacetic acid (EDTA)‐free protease inhibitors (Sigma‐Aldrich). Both orf7a protein constructs were purified according to the protocol of Ref. [36] with minor modifications.

Protein concentration was determined spectrophotometrically in the buffer 20 mM Tris HCl, pH 8.0, 0.15 m NaCl using a molar absorptivity of 7700 m ^−1^ cm^−1^ (for SARS‐CoV‐2 orf7a_*L1*_ and orf7a_*L2*_) and of 1615 m ^−1^ cm^−1^ (for BST2) at 280 nm based on a molecular mass of 10051.22 Da, 9948.08 Da and 14866.61 Da, respectively.

Purity and size of the proteins were checked by sodium dodecyl sulfate‐polyacrylamide gel electrophoresis (SDS‐PAGE) on a pre‐casted NuPage 4–12 % Bis‐Tris polyacrylamide gel (Invitrogen, Carlsbad, CA).

The purified proteins were identified by mass‐spectrometric analysis. After the denaturing SDS‐PAGE run, the proteins of interest were extracted from the gel and analyzed on an Orbitrap FusionTM TribridTM Mass Spectrometer (Thermo) following a tryptic proteolysis (data not shown).

### Sample Preparation for XAS Measurements

Samples for XAS measurements were prepared by adding Zn to each protein solution from the concentrated stock solutions prepared by diluting the ZnSO_4_ stock solution in the buffer 20 mm Tris HCl, pH 8.0, 0.15 M NaCl with 20 % glycerol. The resulting solutions were mixed by pipetting, injected in the sample‐holder used for XAS measurements and immediately frozen in liquid N_2_. Between the preparation and the analysis, samples in their sample‐holders were kept at a temperature close to 77 K, in a dry‐shipper for the shipping^2^.

### Sample Description

In Table 1 we provide a brief description of the samples we have subjected to XAS measurements. In the second column we report the name of the sample, in the third the color we will adopt to draw XAS data curves in the following figures, in the fourth, fifth and sixth we give the concentration of the various proteins present in each sample, in the last column the Zn concentration.

**Table 1:** Protein and Zn concentration in mm of the measured samples.

	Sample name	Color	[BST2]	[orf7a_*L*1_]	[orf7a_*L*2_]	[Zn]
1	BST2	Blue	0.11	–	–	0.1
2	orf7a_*L*1_	Red	–	0.03	–	0.027
3	orf7a_*L*2_	Green	–	–	0.03	0.027
4	BST2_*first*_+orf7a_*L*1_	Purple	0.11	0.03	–	0.1
5	BST2+orf7a_*L*2 *first*_	Orange	0.11	–	0.03	0.1
6	Zn‐buffer	Grey	–	–	–	2

As we already said, the labels *L1* and *L2* appended to the name of the orf7a protein (rows 2 and 3) have been introduced to distinguish between the orf7a constructs differing by the absence (orf7a_*L*1_) or the presence (orf7a_*L*2_) of Cys_15_ at the beginning of the amino acid sequence. The subscript *first* appearing next to BST2 and orf7a_*L*2_ in the rows 4 and 5, respectively, is there to recall that in the case of the sample BST2_*first*_+orf7a_*L*1_, the orf7a_*L*1_ protein was added to an already Zn‐loaded BST2 solution, while, vice versa in the case of the sample BST2+orf7a_*L*2 *first*_, BST2 was added to an already Zn‐loaded orf7a_*L*2_ solution. Note that the Zn concentration has been always taken to be substoichiometric in order to minimize possible spurious XAS signals from free Zn in solution. In the row 6 the concentration of the Zn in buffer is reported.

### XAS Data Collection

XAS measurements on our samples have been carried out at the BM30 beamline of the European Synchrotron Radiation Facility (ESRF – Grenoble, France).^[37]^ The storage ring was operated at an energy of 6 GeV and a beam current of 200 mA, with top‐up refill every hour. The beam energy was selected using a Si(220) double‐crystal monochromator with a resolution of 0.5 eV at the Zn K‐edge. Energy calibration was performed using a Zn metal foil, by setting the first maximum of the first derivative of the XAS spectrum at 9659.0 eV. A Zn metallic foil was located after the samples, in double‐transmission mode, to ensure a calibration of each individual spectrum. The beam spot on the sample was approximately 300×200 μ*m* ^*2*^ (HxV, FWHM). Spectra were recorded in fluorescence mode using a 13‐element solid state Ge detector. To avoid photo‐degradation and spectra evolution during XAS measurements, all the samples, held at 77 K since their preparation, were cooled down and kept at 13 K in a helium cryostat during the whole XAS measurement. For the same reason in the process of data acquisition, samples were systematically moved to a different position after each scan to prevent the beam hitting a previously irradiated spot. Scans never lasted more than 40 minutes. For each sample from 13 to 23 scans, depending on the protein concentration, were merged to obtain average spectra with an acceptable signal‐to‐noise ratio. In the case of the Zn‐buffer, owing to the much higher Zn concentration, average of only 4 scans was enough. XAS spectra were averaged and normalized using the Athena software.^[38]^

### XAS Data

Although we have measured the whole XAS spectrum (XANES and EXAFS regions) of the samples in Table 1, in this paper we will restrict our consideration and analysis to only the XANES region, deferring the study of the EXAFS portion of the spectrum to a forthcoming work.^[26]^

## 2 Results and Discussion

In this section we will discuss the features of the measured XANES spectra and will present some theoretical calculations in support of our analysis.

### 2.1 XANES data

**Figure 3:**
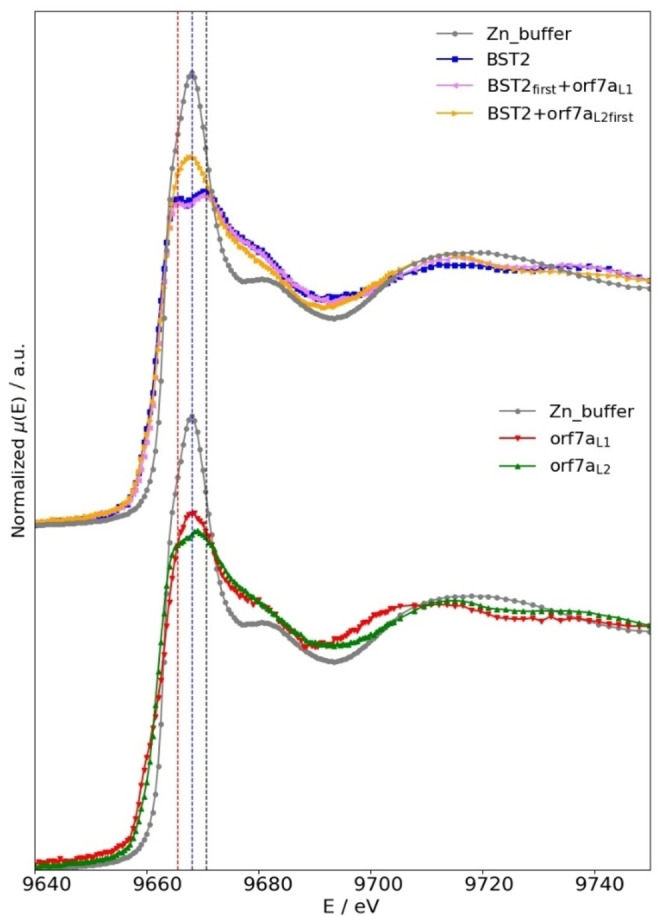
Comparison among the measured XANES spectra. In the top panel we compare the normalized absorption coefficient of the Zn‐buffer (grey curve) to that of BST2 (blue curve), BST2_*first*_+orf7a_*L1*_ (purple curve) and BST2+orf7a_*L2*_ *first* (orange curve) and in the bottom panel to that of orf7a_*L1*_ (red curve) and orf7a_*L2*_ (green curve). The central dotted line is drawn to pass through the maximum of the Zn buffer spectrum at 9668.1 eV, and the other two through the positions of the split maxima of the BST2 spectrum, located at 9665.6 and 9670.6 eV, respectively.

A) In the top panel of Figure 3 we compare the XANES data of the Zn‐buffer (grey curve) to those of BST2 (blue curve), BST2_*first*_+orf7a_*L*1_ (purple curve) and BST2+orf7a_*L*2 *first*_ (orange curve). From this comparison we can make the following observations.

- The XANES spectrum of the Zn‐buffer is significantly different from the spectra of BST2, BST2_*first*_+orf7a_*L*1_ and BST2+orf7a_*L*2 *first*_. This means that BST2 as well as the other two compounds are able to bind Zn. Furthermore, we observe that in the Zn‐buffer spectrum, where the metal is in an octahedral configuration^[39]^ the white‐line peak, located at 9660.8 eV, is more intense and narrow than in the case of the other three samples.^[40]^ We may therefore conjecture that Zn in the BST2, BST2_*first*_+orf7a_*L*1_ and BST2+orf7a_*L*2 *first*_ samples is in a tetrahedral geometry.^[40]^
- There is a double peak at the top of the XANES white‐line of both the BST2 (blue curve) and BST2_*first*_+orf7a_*L*1_ (purple curve) samples. Such a structural feature is typically attributed to the presence of Cys (and possibly His) ligands around Zn in a tetrahedral configuration.^[41]^ The *ab initio* XANES calculations we present in the dedicated section confirm the occurrence of this peculiar behaviour in the spectrum of trial structures where Zn is coordinated to ligand motifs involving Cys and His residues.
- No double peak is featured in the spectrum of the BST2+orf7a_*L*2 *first*_ sample (orange curve), rather a single peak is visible which, however, has a height definitely smaller than that of the Zn‐buffer white‐line again hinting to a tetracoordinated Zn.^[40]^

B) In the bottom panel of Figure 3 we compare the XANES data of the Zn‐ buffer (grey curve) with the spectra of the orf7a_*L*1_ (red curve) and orf7a_*L*2_ (green curve) samples. From this comparison we can make the following observations.

- The XANES spectrum of the Zn‐buffer is significantly different from the spectra of both the orf7a_*L*1_ and orf7a_*L*2_ samples, indicating that Zn is bound to both orf7a constructs.
- The shape of the XANES white‐line of the orf7a_*L2*_ sample shows a sort of two‐fold (or maybe three‐fold) structure which is absent in the case of orf7a_*L*1_. Consistently with what we noticed above we interpret this difference as due to the presence of the extra Cys_15_ as a Zn ligand in the orf7a_*L*2_ sequence which is absent in orf7a_*L*1_ (see the results reported in the “*Ab initio* XAS Calculations” section and Ref. [41]).

C) The picture emerging from the analysis described in A) and B) above is quite interesting.

- First of all, as seen from the top panel of Figure 3, the high similarity of the XANES spectra of the BST2 (blue curve) and BST2_*first*_+orf7a_*L*1_ (purple curve) samples should be interpreted by saying that, once BST2 is loaded with Zn, the addition of orf7a_*L*1_ does not significantly affect the Zn binding mode^3^.

• The XAS spectrum of the BST2+orf7a_*L*2 *first*_ (orange curve) sample is instead appreciably

different from that of BST2 (blue curve) and, as one can see in the bottom panel of

Figure 3, also from that of orf7a_*L2*_ (green curve).

- From this last comparison we conclude that the structure visible in the orf7a_*L2*_ XANES white‐line disappears when BST2 is added. It is tempting to interpret this behaviour by saying that, upon BST2 addition, the Cys_15_ residue of orf7a_*L2*_, to which Zn is supposed to be bound, is made available for the creation of a new disulfide bond with one of the unpaired Cys of the BST2 monomer.

The process illustrated above may be the mechanism behind the formation of the BST2/orf7a complex which, preventing the correct BST2 dimerization, impairs the BST2 antiviral ability.

### 2.2 *Ab initio* XAS Calculations

In order to get some insight as well as quantitative information on the specific way Zn can bind to the BST2 and orf7a proteins, we have performed calculations of the XANES region of the spectrum of models of BST2‐Zn and orf7a‐Zn complexes, assuming that Zn is bound to either Cys's or His's or both in some effective Zn‐finger motif.

For the purpose of this analysis we have made a search in the PDB data bank^[42]^ with the help of the MetalPDB search engine[^43^, ^44^] and identified structures where Zn is coordinated to different numbers of near Cys's and/or His's. The structural models of the trial Zn local structures which we have selected for the XANES calculations are summarized in Table 2. In the insets we display a ball‐and‐stick representation of their atomic configurations. Only one representative atomic structure is displayed not to overload the Table. Since along the PDB structures we have chosen there is often more than one coordinated Zn, in order to unambiguously identify the Zn atom we have selected, we report in parenthesis the progressive number with which the metal is identified in the PDB file. The short‐hand Td and Oh indicate tetrahedral and octahedral coordination, respectively.

**Table 2:** A sketch of the trial Zn structures corresponding to the computed XANES spectra displayed in Figure 4. Color code in the insets is as follows. Zn is in cyan, Oxygen in red, Nitrogen in blue, Carbon in light‐grey, Sulphur in yellow, Hydrogen in green.

	Coordination and Geometry	Schematic Structure	PDB code (Zn atom numbering) and references
1	3 His 1 Cys Td	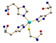	[a] 4ij2 (10870)^[45]^ [b] 2znr (1434)^[46]^ [c] 1v4p (3730)^[47]^
2	2 His 2 Cys Td	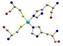	[a] 1rmd (916)^[48]^ [b] 1x6 f (1369)^[40]^ [c] 1a1 h (1164)^[50]^
3	1 His 3 Cys Td	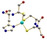	[a] 1btk (2675)^[51]^ [b] 2cup (1514)^[52]^ [c] 1co4(591)^[53]^
4	2 His 1 Asp Td	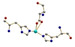	[a]1f5 f (1369)^[54]^ [b] 2nqj(4955)^[55]^
5	1 His 1 Asp (bidentate) 1 water Td	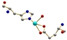	[a]1ci3 (1888)^[56]^ [b] 1 h2b(5389)^[57]^
6	1 His 1 Cys 1 Glu (bidentate) 1 Asp 1 water Oh	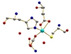	2a21 (4102)^[58]^
7	7 waters Oh	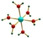

XANES calculations were performed using the FDMNES code.^[59]^ The FDMNES strategy consists in first determining the electronic levels of all the atoms of the cluster in a self‐consistent way. The metal K‐edge XANES spectrum is then computed using the Green method, with muffin‐tin potentials and in the electric quadrupole approximation. This procedure has been already successfully used in Ref. [60] to perform a systematic screening of the XANES spectra of the many Fe‐containing structures available in the Materials Project Library via the Materials Project API.

The theoretical spectra are expressed as function of the photo‐electron energy. To compare them with the experimental spectra and convert photo‐electron energy in incident photon energy, a constant energy value of 9659.3 eV was accounted for in defining the photo‐electron energy. This value was optimized to allow the best energy alignment of the Zn‐buffer white‐line position of both the calculated and experimental spectra. This correction was made in all the spectra.

**Figure 4:**
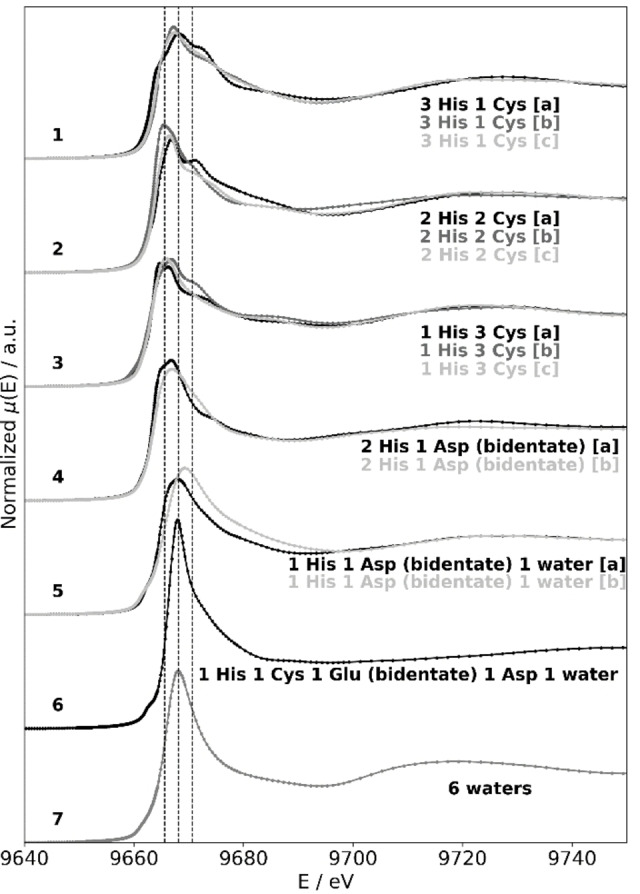
The computed XANES spectra of the Zn complexes listed in Table 2.

The results collected in Figure 4 show that peculiar features appear at the top of the XANES white‐line in Zn‐structures where the metal is tetracoordinated to 3Cys‐1His, 2Cys‐2His or 1Cys‐3His (first three sets of curves in Figure 4). In each one of these three cases we performed three separate calculations of the XANES spectrum starting from the three independent structures labelled by [a], [b] and [c] in the figure and correspondingly in Table 2. In all the cases the presence of a structured white‐line with peaks and dips is confirmed. The too small differences encountered in the XANES computation of almost similar structures (*i. e*. structures that have the same number of Zn‐coordinated Cys and His) do not allow to push further the interpretation of the calculations and determine exactly what is the Zn local structure.

The calculations of the XANES spectrum of structures where Zn in a tetrahedral geometry is surrounded by His ligands and possibly water, but not by Cys (like in the situation we have in the case of 2 His and 1 His structures in the curves labelled by 4 and 5 in Figure 4, see also rows 4 and 5 of Table 2) show that just a single smooth peak is observed in the white‐line, with a significantly smaller height compared to that of the Zn‐buffer spectrum, and no shoulder around 9690 eV. These spectral features fit well with the shape of the white‐line of the BST2_*first*_+orf7a_*L*1_ and orf7a_*L*1_ samples (orange and red curves in Figure 3). Similarly, no structure is visible in the white‐line in case Zn is in an octahedral coordination like in the case of the curves labelled by 4 and 5 in Figure 4, corresponding to the samples displayed in rows 6 and 7 of Table 2.

Summarizing this discussion we can say that our extensive calculations show that the characteristic double peak structure at the top of the XANES white‐line visible in the spectra of BST2 (red and blue curves in the top panel of Figure 3) and orf7a_*L*2_ (green curve in the bottom panel of Figure 3) is the result of a specific Zn−Cys/His interaction with the metal in a tetrahedral geometry. This is confirmed by the fact that in the absence of cysteine (curves 4 and 5) or when Zn is in an octahedral coordination (curves 6 and 7) these peculiar spectral features are not borne out by theoretical calculations.

## 3 Conclusions

The work we have presented in this paper is the first XAS investigation of the BST2‐Zn, orf7a‐Zn and BST2‐orf7a‐Zn complexes. The main conclusions of our structural study are as follows.

1. As one can see from the top panel of Figure 3, the XANES spectrum of the Zn‐buffer (grey curve) is significantly different from that of BST2 (blue curve). This is telling us that BST2 is able to bind Zn. In particular, as discussed in refs.[^34^, ^41^] and confirmed by the *ab initio* XANES calculations presented in sect. “*Ab initio* XAS Calculations”, we interpret the peculiar double peak feature visible at the top of the XANES white‐line as due Zn binding the BST2 protein via some of the Cys/His motifs present along the amino acid sequence.
2. In the bottom panel of Figure 3 we compare the XANES spectrum of the Zn‐buffer with the spectra of orf7a_*L*1_ (red curve) and orf7a_*L*2_ (green curve). The comparison shows that orf7a, in both its constructs (either 16–81 or 15–81), is able to bind Zn. Moreover one should notice the significant difference in the shape of the white‐line around its maximum between the green and the red curve. This difference, according to the results of our XANES calculations (see the “*Ab initio* XAS Calculations” section), should be ascribed to the presence of the extra Cys_15_ Zn‐ligand in orf7a_*L*2_.
3. Comparing now the spectrum of orf7a_*L*2_ (green curve in the bottom panel of Figure 3) with the spectrum of BST2+orf7a_*L*2 *first*_ (orange curve in the top panel of Figure 3), we see that the structure visible at the top of the white‐line of the XANES region of orf7a_*L*2_ is absent in the case of BST2+orf7a_*L*2 *first*_. We interpret the surprising disappearance of the double‐peak structure when BST2 is added to an already Zn‐loaded orf7a_*L2*_ sample, by saying that in the presence of BST2, the Cys_15_ amino acid of orf7aL2, upon losing the metal, it is made available for the creation of a disulfide bond across the two proteins leading to the formation of BST2/orf7a complexes. Therefore, the virus ability to counteract the BST2 antiviral activity could be the result of the formation of the BST2/orf7a complex.
4. On the contrary, in the situation where orf7a_*L1*_ (which lacks the Cys_15_ ligand) is added to an already Zn‐loaded BST2 solution (purple curve in the top panel of Figure 3), the persistence of the double peak feature at the top of the XANES white‐line (absent in the case of orf7a_*L1*_ alone, as seen in the smoother shape of the red curve in the bottom panel of Figure 3), suggests that orf7a_*L1*_ is unable to modify the Zn‐BST2 coordination mode and bind the metal, at least at the concentrations we are working at. This is consistent with the pivotal role of Cys_15_ as a Zn‐ligand that the interpretation of our data implies.
5. Extensive *ab initio* XANES calculations confirm that the structure visible at the top of the white‐line, with its peaks and dips, occurs if Zn in a tetrahedral configuration is coordinated to some Cys/His motif where, together with His's, at least one Cys is present (see Figure 4).

This work is a first step in the direction of understanding the role of Zn in the interactions between BST2 and some of the accessory SARS‐CoV‐2 proteins. We have shown that both the host BST2 protein and the viral orf7a protein are able to bind Zn. Our analysis supports the hypothesis that BST2/orf7a complexes get formed with the creation of a disulfide bond pairing the Cys_15_ amino acid of orf7a with some of the available Cys along the BST2 chain.

To obtain a more accurate picture of where Zn can possibly bind, one needs to extend the present study to include the structural information that can be extracted from the analysis of the EXAFS region of the spectrum. An investigation where the BST2 and orf7a EXAFS data will be employed to identify and more precisely characterize possible Zn binding sites along the two protein sequences is currently in progress.^[26]^ The construction of reliable atomic models of the Zn geometrical environment requires extensive Molecular Dynamics simulations and, wherever possible, *ab initio* DFT calculations which both will be addressed.

Naturally, a crucial step in the direction of elucidating the alleged inhibition of the BST2 virion immobilization ability operated by accessory proteins is to provide good XAS data also for the orf8 protein. Until today this has not been possible owing to the difficulty of producing a sufficiently large amount of purified orf8 protein. We are confident, however, that we will soon be able to overcome the problem.

## Conflict of interest

The authors declare no conflict of interest.

## Footnotes

n1 The amino acid sequences of the segments of the orf7a protein^[5]^ we consider here are orf7a_*L1*_ (16‐82) ELYHY QECVRGTTVL LKEPCSSGTY EGNSPFHPLA DNKFALTCFS TQFAFACPDG VKHVYQLRAR SV. orf7a_*L*2_ (15‐82) CELYHY QECVRGTTVL LKEPCSSGTY EGNSPFHPLA DNKFALTCFS TQFAFACPDG VKHVYQLRAR SV. The amino acid sequence of BST2^[35]^ is MASTSYDYCR VPMEDGDKRC KLLLGIGILV LLIIVILGVP LIIFTIKANS EACRDGLRAV MECRNVTHLL QQELTEAQKG FQDVEAQAAT CNHTVMALMA SLDAEKAQGQ KKVEELEGEI TTLNHKLQDA SAEVERLRRE NQVLSVRIAD KKYYPSSQDS SSAAAPQLLI VLLGLSALLQ. We have outlined in red the Cysteine and in blue the Histidine residues. Do we have colours in the text of the printed paper? If not please erase the underlined sentence in black.

n2 Because of the restrictions due to the pandemic, samples prepared in Rome were shipped to Grenoble where experiments have been performed in remote mode.

n3 Actually to complete the analysis it would have been desirable to also measure the XAS spectrum of a sample where to the Zn‐loaded BST2, the orf7a_*L*2_ construct is added to see whether the presence of the key Cys_15_ residue could alter the pre‐existing BST2‐Zn binding mode.
